# Antimicrobial Resistance Profile of *Pseudomonas aeruginosa* from Different Clinical Samples in Debre Tabor Comprehensive Specialized Hospital, Northwest Ethiopia

**DOI:** 10.4314/ejhs.v33i3.5

**Published:** 2023-05

**Authors:** Tsigereda Asamenew, Seble Worku, Hilina Motbainor, Daniel Mekonnen, Awoke Deribe

**Affiliations:** 1 Department of Medical Microbiology, College of Medicine and Health Sciences, Bahir Dar University, Bahir Dar, Ethiopia; 2 Department of Medical Laboratory Sciences, College of Medicine and Health Sciences, Debre Tabor University, Debre Tabor, Ethiopia; 3 Department of Health Biotechnology, Biotechnology Research Institute, Bahir Dar University, Bahir Dar, Ethiopia; 4 Centre for Innovative Drug Development and Therapeutic Trials for Africa (CDT-Africa), Addis Ababa University, Addis Ababa, Ethiopia*

**Keywords:** Pseudomonas aeruginosa, Antimicrobial susceptibility profile, Debre Tabor

## Abstract

**Background:**

Pseudomonas aeruginosa is one of the leading causes of hospital-acquired infections and the most common antimicrobial-resistant pathogens. It is associated with a variety of infections. This study aimed to determine the prevalence of P. aeruginosa and its antimicrobial resistance profile from different clinical specimens at Debre Tabor Comprehensive Referral Hospital (DTCRH).

**Methods:**

A cross-sectional study was conducted from May to July 2022 at DTCRH. Socio-demographic and clinical data were collected using a structured questionnaire. Clinical samples (blood, wound swab, urine, and sputum) were collected from 348 study participants and processed following the standard bacteriological techniques. Antibiotic susceptibility testing was done by the Kirby-Bauer disc diffusion method. Data were entered and analyzed using SPSS version 25 statistical software. Descriptive statistics was used to present the findings of the study.

**Results:**

The prevalence of P.aeruginosa was 74(19.3%). The detection of the isolates was different based on the type of samples that ranged from 0% to 54.5% from sputum and wound swabs, respectively. P.aeruginosa showed resistance against gentamicin at 62.2%, ceftazidime 51.4%, cefepime 50%, amikacin 29.7%, imipenem 28.4% and ciprofloxacin 14.9%. The level of multi-drug resistance (MDR) was 45.9%, and the suspicious extreme-drug resistance (XDR) rate was 9.5%. Being inpatient and wound swab samples were factors associated with the detection of P.aeruginosa from clinical samples.

**Conclusion:**

The antibiotic resistance profile of P. aeruginosa isolates in the present study area was found to be alarming. Actions to minimize the effect of antimicrobial resistance should be strengthened, and further large-scale study should be conducted to find out the main reasons behind antibiotic resistance of P.aeruginosa and other clinically relevant isolates.

## Introduction

*P. aeruginosa* is one of the most common causes of different bacterial infections globally. The prevalence of the isolate was higher (13.2–22.6%) in intensive care units (ICUs), in patients with cystic fibrosis, pneumonia, urinary tract infections, burn, and wound infections (1).

In developing countries, the prevalence of hospital-acquired infections (HAI) due to *P. aeruginosa* is higher, especially among patients in ICUs (2). A meta-analysis report showed that the pooled proportion of *P.aeruginosa* in different clinical specimens (like urine, wound, blood and other body fluids) in Africa was 21.4% (3).

*P.aeruginosa* commonly causes infections in individuals with a damaged outer barrier of the skin following the burn, surgery, and accidental tissue damage, at the insertion site of a catheter or endotracheal tubes and scratched cornea. Immune-compromised patients with HIV, diabetes, and cancer are also more vulnerable to developing infections (4). The spread of these infections in hospital settings is mostly related to a lack of adherence to infection prevention and control protocols (5). Evidence showed that the prevalence and magnitude of drug-resistant *P. aeruginosa* isolates were higher in developing countries. The pathogen is becoming multi-drug resistant (MDR) due to different intrinsic and extrinsic factors making the treatment of common infections difficult (6).

In Ethiopia, studies were conducted sporadically at different places to demonstrate the prevalence of *P.aeruginosa* and its drug resistance pattern. According to a study by Bitew et al., for example, the prevalence of *P. aeruginosa* from different clinical samples was 7.8% in Ethiopia (7). Along with different reports, there was an increasing prevalence of nosocomial infections due to *P.aeruginosa* and high drug resistance including MDR conditions (7–11). Low socio-economic statuses (poverty, resource scarcity, and poor rational antimicrobial usage) were significantly associated with the transmission and drug resistance of nosocomial pathogens (8).

Most of the similar studies in Ethiopia were conducted on a specific specimen and population. Moreover, antibiotic resistance patterns are different geographically and change over time. Hence, this study aimed to determine the prevalence of *P.aeruginosa* and its antimicrobial resistance profile from different clinical specimens at Debre Tabor Comprehensive Referral Hospital (DTCRH).

## Materials and Methods

**Study design and population**: A hospital-based cross-sectional study was conducted from 10 May to 15 July 2022 in DTCRH. The hospital is located in Amhara Regional State, Northwest Ethiopia, which has been serving more than 2 million people.

All patients who had bacteriological culture and antimicrobial susceptibility test requests from blood, wound swab, urine, and sputum samples during the study period were included. At the time of data collection, patients who had a history of antibiotic treatment in less than two weeks were excluded from the study (12). A total of 384 study participants were included in the study consecutively until the required sample size was achieved. The sample size was calculated based on a single population proportion formula by taking the prevalence of *P. aeruginosa* from different clinical samples at 49.3% (0.49) which was reported by Bekele et al. in Jimma University Hospital, Ethiopia (9).

**Data collection**: Socio-demographic data like the participants' age, sex, educational status, occupation, and residence were collected using an interview-based structured questionnaire. Likewise, clinical-related data such as admission history, patient visits (outpatient/inpatient), and antimicrobial drug use were collected using the questionnaire.

**Blood sample collection and processing**: About 10ml, 5ml, and 2ml of venous blood samples were collected from adults, children, and neonates, respectively, following aseptic techniques. Prior to sample collection, the sample collection area was cleaned with 70% ethanol and an iodine-based solution to further prevent microorganisms from contaminating the blood sample. After collection, the blood was dispensed to Tryptic Soya broth medium (Oxoid, England) in duplicates, then incubated aerobically at 35-37°C, and bacterial growth was inspected daily. When there was any growth observed during the daily inspection, gram staining and sub-culture was done on to MacConkey (MAC) agar and blood agar (BA) (Oxoid, England). Cetrimide Agar was used as a selective medium. Blood culture was reported as negative after seven days of no growth in the bottle and subcultures.

**Wound swab sample collection and processing**: Wound/pus samples were collected by using sterile cotton swabs dipped in normal saline, and the swab was kept in a sterile test tube. The collected specimen was transported immediately to the DTCRH microbiology laboratory for analysis. The sample was inoculated on MacConkey agar, blood agar plate, and Cetrimide Agar and incubated at 35-37°C overnight aerobically. Identification of the isolates was performed using colony morphology, Gram-staining, and conventional biochemical tests (13).

**Urine sample collection and processing**: Participants were instructed to collect approximately 5 to 10ml of midstream clean-catch urine by using a wide-mouthed sterile sample container. For catheterized participants, samples were collected by using a sterile 10 ml syringe from the storage bag through the sampling port after cleaning the area by using 70% alcohol. Then, the sample was transferred to a sterile cup and transported to the DTCRH microbiology laboratory. The samples were inoculated by using 1ul containing loop on MaConckey, blood agar, and Cetrimide Agar and were incubated at 35-37 °C aerobically overnight. Significant bacteriuria was declared when the number of colony-forming units (CFU) per ml of urine was ≥10^2^ for catheterized samples and ≥10^5^ for clean catch samples (10).

**Sputum sample collection and processing**: Deeply coughed early morning sputum samples were collected using a wide-mouthed screw cup after instructing the participant how to collect the sample. Then, the samples were transported to the DTCRH microbiology laboratory for bacteriological culture analysis like the other samples described above.

**Identification of *P.aeruginosa***: Identification of *P.aeruginosa* was done based on colony morphology, gram reaction, and different panels of biochemical tests. It was presumptively identified based on its large, flat and dark greenish colonies on blood agar and non-lactose fermenter pale colonies on MacConkey agar. Besides, cetrimide agar was also used for *P.aeruginosa* identification. Common biochemical tests including oxidase, catalase, citrate utilization, and oxidative fermentative were also done (13).

**Antimicrobial susceptibility testing**: Antimicrobial susceptibility testing was performed using the disk-diffusion method following the Clinical and Laboratory Standards Institute (CLSI) guideline.

Briefly, using a sterile wire loop 3-5 similar pure colonies were picked from MacConkey agar and homogenized in nutrient broth. The colony concentration was adjusted based on the 0.5 McFarland standards, and the inoculum was swabbed on the Muller Hilton agar (100mm plate) by using sterile cotton swabs.

The following antibiotic discs were used to test the antimicrobial resistance profile of *P. aeruginosa*: Cefepime (30µg), Ceftazidime (30µg), Imipenem (10µg), Gentamicin (10 µg), Ciprofloxacin (5µg), and amikacin (30µg). The antibiotic discs were placed on Muller-Hilton agar, which are previously inoculated with test strains and incubated at 37 °C for 16–18hrs. Then, the zone of inhibition around the antimicrobial disc was measured and interpretation was done based on the CLSI protocol (14, 15).

MDR was defined as the resistance of *P. aeruginosa* to three or more antimicrobial classes while XDR was non-susceptibility to at least one agent in all but two or fewer antimicrobial classes (i.e. *P. aeruginosa* remains susceptible to only one or two antimicrobial classes).

**Data quality assurance**: Standard Operating Procedures (SOP) were strictly followed during the sample collection and processing steps. The performance and sterility of culture media, biochemical tests, and discs for AST were tested by using *P.aeruginosa* ATCC 27853 standard control strains.

**Data organization, processing, and analysis**: Data were entered and analyzed using SPSS version 25 software. The results were presented through tables and figures. Descriptive statistics was used to summarize the isolation and susceptibility profile of *P. aeruginosa*. Logistic regression analysis was carried out to identify factors associated with *P.aeruginosa* isolates from clinical specimens. In bivariable analysis, variables with p-value <0.2 were entered into the multivariable analysis. p-value <0.05 was considered statistically significant.

**Ethical issues**: Ethical clearance was obtained from Bahir Dar University, College of Medicine and Health Science, Institutional Ethical Review Board with reference number 389/2022. Written informed consent was obtained from all participants after the purpose and objectives of the study were explained. The study subjects were informed that all the data and samples obtained from them would be kept confidential. For each culture-confirmed case, not only *P. aeruginosa* but also any other medically important isolates, the result was communicated to the clinician in charge of attending to the respective patients.

## Results

**Socio-demographic and clinical characteristics of study participants**: A total of 384 participants were included in this study. Out of these, 238 (62%) were males. The mean ±SD age of the participants was 35.36 ±16.7 years. The majority of the study participants, 152 (39.6%), were in the age group of 18-30 years. Data on educational status showed that 184(47.9%) had no formal education. Similarly, almost half of the participants (48.2%) were previously admitted at least once for medical attention. Further, most of the participants (58.1%) were attending service in the outpatient department, and about 25.3% had known chronic illnesses.

Among the 384 participants, 159(41.4%) urine, 112(29.2%) pus/wound swabs, 73(19%) sputum, and 40(10.4%) blood samples were collected, for bacteriological analysis (Table 1).

**Table 1:** Socio-demographic and clinical characteristics of study participants at DTCRH, 2022

Variables	Category	Frequency	Percent
Gender	Male	238	62.0
	Female	146	38.0
Age group in years	<18	46	12.0
	18-30	152	39.6
	31-40	68	17.7
	41-60	84	21.9
	>60	34	8.9
	Others	10	2.6
Educational status	Has no formal education	184	47.9
	Primary school	88	22.9
	Secondary school	49	12.8
	University/College	63	16.4
Residence	Urban	160	41.7
	Rural	224	58.3
Previous admission history	Yes	185	48.2
	No	199	51.8
Patient status	Outpatient	223	58.1
	Inpatient	161	41.9
Known chronic disease	Yes	97	25.3
condition	No	287	74.7
Type of the chronic disease	Hypertension	15	3.9
	Diabetes mellitus	66	17.2
	Epilepsy	7	1.8
	Cardiac disease	1	.3
	HIV/AIDS	8	2.1
Type of specimen collected	Urine	159	41.4
	Pus/wound swab	112	29.2
	Sputum	73	19.0
	Blood	40	10.4

**Prevalence of *P.aeruginosa***: Of the 384 clinical samples processed, 74(19.3%) were found positive for *P.aeruginosa. P.aeruginosa* was recovered from urine, blood, and wound swab at 9(5.7%),4(10%), and 61(54.5%), respectively. No isolation was recorded from sputum samples (Table 2).

**Table 2:** Distribution of *P. aeruginosa* in urine, blood, sputum, and wound swab at DTCRH, 2022

	Types of specimens			
	
The isolate	Urine	Blood	Sputum	Pus/wound swab
	N (%)	N (%)	N (%)	N (%)
*P. aeruginosa*	9 (5.7)	4 (10)	0(0)	61(54.5)
No growth	150 (94.3)	36 (90.0)	73 (100)	51 (45.5)
**Total**	159 (100)	40 (100)	73 (100)	112 (100)

**Figure 1:**
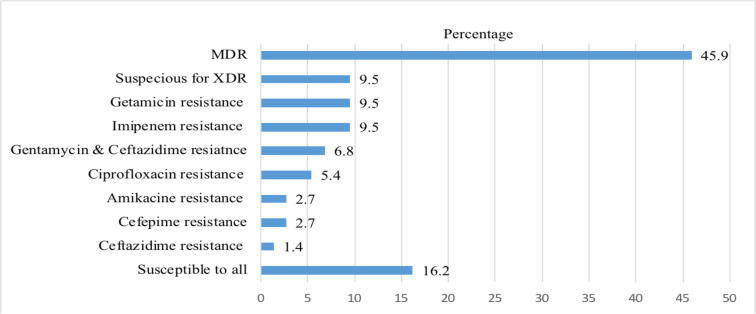
Antibiotic resistance profile of P. aeruginosa at DTCRH, 2022.

**Antimicrobial resistance profile of *P.aeruginosa*:** The antimicrobial resistance profile of *P.aeruginosa* was interpreted based on the CLSI guideline. The AST result showed that 46(62.2%) and 22(29.7%) of *P.aeruginosa* isolates were found to be resistant to gentamicin and amikacin, respectively. The lowest antibiotic resistance was recorded against ciprofloxacin, at 11(14.9%). The third generation Cephalosporins (ceftazidime) and fourth generation Cephalosporins (Cefipime) had almost comparable resistance patterns against *P.aeruginosa*, at 38 (51.4%) and 37(50%), respectively (Table 3). On top of this, the level of multi-drug resistance (MDR) and XDR was 45.9% and 9.5 % of, respectively (Figure 1).

**Table 3:** Antimicrobial susceptibility profile of *P. aeruginosa* at DTCRH, 2022

Classes of antibiotics	Type of antimicrobials	Susceptible N (%)	Resistant N (%)	Intermediate N (%)
**Aminoglycoside**	Gentamicin (10µg)	28 (37.8)	46 (62.2)	0 (0)
**Fluoroquinolones**	Ciprofloxacin (5µg)	34 (45.9)	11 (14.9)	29 (39.2)
**Cephalosporin**	Ceftazidime (30µg)	36 (48.6)	38 (51.4)	0 (0)
**Aminoglycoside**	Amikacin (30µg)	46 (62.2)	22 (29.7)	6 (8.1)
**Cephalosporin**	Cefepime (30 µg)	37 (50.0)	37 (50.0)	0 (0)
**Carbapenem**	Imipenem (10µg)	51 (68.9)	21 (28.4)	2 (2.7)

**Factors associated with the detection of *P.aeruginosa***: The multivariable regression analysis showed that the detection of *P.aeruginosa* was more than six times more likely among the inpatients compared to the outpatients (AOR=6.64; 95%CI; 3.67-11.99; p<0.001). Similarly, *P.aeruginosa* was detected 16 times more likely (AOR: 16.7; 95%CI; 5.98-36.26, p<0.001) from wound samples compared to the rest of the clinical specimens. The rest of the variables (gender, occupation, and having known chronic illness) did not show a statistical association (Table 4).

**Table 4:** Factors associated with the detection of *P. aeruginosa* at DTCRH, 2022

Variables	Category	P. aeruginosa			
		
		Positive N (%)	Negative N (%)	AOR (95% CI)	*p-value*
**Gender**	Male	51 (21.4)	187 (78.6)	0.467 (0.19-1.13)	0.142
	Female	23 (15.8)	123 (84.2)	1	
**Occupation**	Unemployed	8 (11.3)	63 (88.7)	1.82 (0.193-17.16)	0.600
	Full-time employed	9 (12.5)	63 (87.5)	0.38 (0.038-3.877)	0.418
	Daily worker	1 (33.3)	2 (66.7)	0.117 (0.003-4.28)	0.243
	Merchant	8 (16.7)	40 (83.3)	0.745 (0.075-7.38)	0.802
	Farmer	46 (25.6)	134 (74.4)	1.21 (0.123-11.97)	0.869
	Others	2 (20.0)	8 (80.0)	1	
**Patient status**	Inpatient	57 (35.4)	104 (64.6)	6.64 (3.67-11.99)	<0.001
	Out patient	17 (7.6)	206 (92.4)	1	
**Known chronic**	Yes	15 (15.5)	82 (84.5)	1.42 (0.76-2.63)	0.273
**disease condition**	No	59 (20.6)	228 (79.4)	1	
**Specimen type**	Urine	9 (5.7)	150 (94.3)	0.54 (0.157-1.85)	0.327
	Sputum	0 (0.0)	73 (100.0)	-	-
	Pus/wound infection	61(54.5)	51 (45.5)	16.67(5.98-36.26)	<0.001
	Blood	4 (10.0)	36 (90.0)	1	

AOR: Adjusted Odds Ratio

## Discussion

*P. aeruginosa* is one the most common causes of healthcare-associated infections throughout the world with ongoing drug resistance records. It remains as the main public health concern, especially in resource-limited settings where the problem of drug resistance impacts millions. Similar to the present study, multiple clinical samples including wound swabs, sputum, and urine were used to determine the prevalence and drug susceptibility patterns of *P .aeruginosa* (16) but with inconsistent reports.

In this study, the prevalence of *P. aeruginosa* from the four clinical samples was 19.3%. Comparable findings at 16.4-19.4% were reported in different parts of the world (17–20). On the contrary, in a multi-center study by Restrepo et.al, a low proportion of *P. aeruginosa* at 4.2% was documented (21). Likewise, studies in Ethiopia reported a 7.1-11.9% proportion of *P. aeruginosa* from different clinical samples (22, 23). However, a higher prevalence *P. aeruginosa* was also reported in Jimma University Hospital, Ethiopia (49.32%) (9). Differences in the reported figures might be due to a number of reasons, including the type and severity of infections, the type of enrolled study population, and the sample size. In the present study, no *P. aeruginosa* was isolated in sputum, and it was low in blood samples as well. This is in agreement with a multi-center study that reported about 4% (21).

In our study, the antimicrobial resistance profile of *P. aeruginosa* was tested, and a high resistance rate was documented against gentamicin (62.2%), ceftazidime (51.4%), and cefepime (50%). Medium resistance was reported for amikacin (29.7%) and imipenem (28.4%). Comparable results were reported in Ethiopia and elsewhere in the world. For example, in China, 51.1% resistance for gentamicin and 22.2% level of resistance for Amikacin (17) were reported against *P. aeruginosa*. Likewise, a concordance result was reported in Iran where resistance for amikacin and ciprofloxacin were 23% and 19.5%, respectively (24). Studies in India reported 51%, 24%, and 23.1% of resistance against gentamicin, amikacin, and imipenem, respectively (16). On the contrary, a study in Spain showed that the resistance of *P. aeruginosa* was 8%, 7%, 3%, 1%, and 0% for ceftazidime, cefepime, gentamicin, ciprofloxacin, imipenem, and amikacin, respectively (25). The high resistance patterns of the isolate for different classes of antimicrobials in different parts of the world call a collaborative action to minimize the effect of antimicrobial resistance (26,27).

In our study, multi-drug resistant (MDR) and suspicious to extensive drug-resistant (XDR) isolates were also identified. The rate of MDR was 45.9%, which indicated that almost half of the *P.aeruginosa* isolates were resistant to at least three different classes of antibiotics, and about 9.5% of the isolates were found suspicious to XDR. Similar MDR findings were reported in Ethiopia at 50% (11). On the other hand, low rates of MDR were reported in USA (14%) (28), Spain (5.5%) (25), and Pakistan (30%) (27). The result indicated that the MDR profile of *P.aeruginosa* was very high in this study, which might be due to multiple factors including the common routine of prescribing broad-spectrum antibiotics without any proper laboratory identification like, culture and drug susceptibility test mainly due to the inaccessibility of microbiological laboratories. Poor rational drug use, poor knowledge of antimicrobial usage in the community, poor sanitation practices, and irrational prescriptions could be additional factors for the escalation of MDR (29).

The detection of *P.aeruginosa* from clinical samples was affected by different factors. In this study, a higher rate of *P.aeruginosa* isolation was observed in inpatient participants compared to outpatient clients. Similarly, the rate of *P. aeruginosa* was more than 16 times more likely to happen in wound swabs compared to the blood sample. A similar finding was reported in India where there was a higher detection rate of *P. aeruginosa* from wound swabs compared to the other samples (16). Although *P. aeruginosa* was one of the most common causes of nosocomial infections and mostly affected immunocompromised patients (30). In this study, living with chronic disease conditions specifically hypertension, diabetes mellitus, HIV AIDS, and cardiac disease were not statistically associated with the detection of *P. aeruginosa*.

We used only a few antimicrobial discs for the antimicrobial susceptibility testing due to resource limitations, which might compromise to appreciate the full antimicrobial resistance picture of the isolate.

In conclusion, the prevalence of *P. aeruginosa* was 19.3% from urine, wound swabs, and blood. A high prevalence of *P.aeruginosa* was identified from wound swabs at 54.5%. In this study, the rate of MDR was at 45.9%. The detection of *P.aeruginosa* was statistically associated with the participants being an inpatient and the sample being a wound swab. Even though the prevalence of *P.aeruginosa* was relatively medium compared to the previous similar studies in Ethiopia, the recorded antimicrobial resistance profile was very alarming. Therefore, actions to minimize the effect of antimicrobial resistance should be strengthened, and further large-scale studies should be conducted to find out the main reasons behind antibiotic resistance of *P. aeruginosa* and other clinically relevant isolates in the study area.
